# Early Implanon discontinuation and associated factors among Implanon women users visiting public health facilities, in Kembata zone of Southern Ethiopia: An institution based cross-sectional study

**DOI:** 10.3389/fgwh.2022.909411

**Published:** 2022-08-11

**Authors:** Gemechu Nigusie Beyene, Nega Assefa, Taklu Marama Mokonnon, Habtamu Bekele Ejigu, Tesfaye Assebe Yadeta

**Affiliations:** ^1^Doyogena Primary Hospital, Doyogena, Ethiopia; ^2^School of Nursing and Midwifery, College of Health and Medical Science, Haramaya University, Harar, Ethiopia; ^3^School of Midwifery, College of Health Sciences and Medicine, Wolaita Sodo University, Sodo, Ethiopia

**Keywords:** Implanon, discontinuation, women, Ethiopia, early

## Abstract

**Background:**

Contraceptive practice is the basis of fertility and plays a significant role in minimizing maternal morbidity and mortality. Implanon is one of the most effective long-acting contraceptive methods that prevents pregnancy for 3 years. Early Implanon discontinuation can lead to many negative reproductive health consequences. Therefore, this study assessed the magnitude of early Implanon discontinuation among female Implanon users visiting public health facilities to discontinue Implanon in the Kembata zone of southern Ethiopia.

**Methods:**

An institution-based cross-sectional study design was carried out from 1 March 2020 to 30 March 2020. Simple random sampling techniques were used to select 454 women who had discontinued Implanon. The data were collected using a pretested and structured questionnaire. The collected data were entered into Epi Data version-3.1 and then exported to STATA version-16 for analysis. A descriptive analysis along with bivariate and multivariate regression was performed to identify factors associated with early Implanon discontinuation. Statistical significance was declared at a *p* < 0.05 along with 95% confidence intervals (CIs).

**Results:**

In the present study, 438 women were interviewed, which corresponds to a response rate of 96.48%. The magnitude of early Implanon discontinuation was 56.4%, 95%CI (51.6, 61.2). Primary educational status [adjusted odds ratio (AOR) = 2.92, 95%CI (1.56, 5.46)], rural residency [AOR = 1.76, 95%CI (1.06, 2.92)], women with no history of modern contraceptive use [(AOR = 2.14, 95%CI (1.14, 4.03)], those who experienced service dissatisfaction [AOR = 3.05, 95%CI (1.52, 6.12)], women who experienced as Implanon side effect [AOR = 3.36, 95%CI (2.09, 5.42)], and women who were not appointed after insertion [(AOR = 2.17, 95%CI (1.18, 4.79)] have shown an association with early Implanon discontinuation.

**Conclusion:**

The present study indicated that the magnitude of early Implanon discontinuation was high. Educational level, rural residency, women who experienced side effects, those who experienced service dissatisfaction, women with no history of contraceptive use, and those who missed follow-up appointments were associated with Implanon discontinuation. Family planning service providers should focus on Implanon side effects during pre-insertion counseling. The recommended intervention is to recognize modifiable factors like improving client satisfaction with the service, appointing for follow up after insertion, and providing quality family planning services.

## Introduction

Contraceptive use is the basis of fertility, which plays a significant role in minimizing maternal morbidity and mortality (1). Implanon is one of the long-acting reversible contraceptives that prevent pregnancy for 3 consecutive years with a failure rate of <1% (2). More than 4.5 million women worldwide have used Implanon (3, 4). In sub-Saharan Africa, an increasing number of women are using contraceptive implants. In Ethiopia, around 8% of women in the reproductive age group are using implants (5, 6). Even though new women in the reproductive age group are enrolling in the use of Implanon, the discontinuation rate of Implanon before the due date is alarming. Studies conducted in different corners of Africa, including Ethiopia, showed that 17–47% of women who received Implanon discontinued before the expected removal date (7–9).

Nowadays, the problem of contraceptive discontinuation without method switching is a major public health concern because it is associated with negative reproductive health outcomes such as unplanned childbearing and unintended pregnancies leading to unsafe abortions. This contributes a lot to maternal morbidity and mortality (10, 11). The burden of unintended pregnancies in women in the reproductive age group is highest in Latin America (96 per 1,000), followed by Africa (89 per 1,000 women) (12). This might be due to the lack of access to contraceptive methods or contraceptive discontinuation without method switching (13).

Despite the commitment of the Ethiopian Ministry of Health over the past few decades to promoting reproductive services where contraceptive continuation is a priority, the discontinuation of Implanon is unacceptably high (14). This might be linked to various factors like the lack of emphasis on the side effects of Implanon during counseling, the lack of spousal communication to use contraceptive methods, the quality of services provided, and a need to have a child in the future (15, 16).

Addressing the factors that contribute to early Implanon discontinuation is essential to ensure safe and reliable services to women in the reproductive age group through proper use of contraceptive methods and method switching. Even though studies to delineate the magnitude of Implanon discontinuation have been conducted in some countries, it is still an ongoing problem in the study area. Data on early Implanon discontinuation are also sparse in the study area. In addition, the epidemiologically reliable magnitude and factors associated with early Implanon discontinuation, given the appropriate sample size, have not been well-studied. The findings of this study will boost the planning and decision-making capabilities of healthcare professionals in seeking possible solutions for clients in collaboration with concerned stakeholders who work on family planning programs. Therefore, the present study assessed the magnitude of early Implanon discontinuation and associated factors among female Implanon users visiting public health facilities in the Kambata Tambaro zone of southern Ethiopia in 2020.

## Methods

### Study period, design, and setting

An institution-based cross-sectional study design was conducted from 1 March 2020 to 30 March 2020. The Kambata Tambaro zone is located in the Southern Nation Nationalities and People Regional State and its administrative town is called Durame. Durame is located 306 km away from Addis Ababa, in the Southwest direction. Based on the 2007 National Census conducted by the Central Statistical Agency of Ethiopia (CSA), the zone's current total population is 941,313, of which 480,070 are women and 219,326 of them are in the reproductive age group (15–49). Around 175,460 are eligible for family planning, and 14,622 are expected to use Implanon in the 2012 Ethiopian Fiscal Year (17).

### Population and eligibility criteria

All women who attended health facilities to discontinue Implanon in the Kambata Tambaro zone were the source population of this study. The study included women who were residents of the study area in the last 6 months and visited selected public health facilities during the data collection period to discontinue Implanon. Women who were seriously ill were excluded.

### Sample size determination and sampling procedure

The required sample size was calculated using a single population proportion formula, taking into account the following assumptions from the study conducted in southern Ethiopia: a confidence interval (CI) of 95%, a margin of error of 5%, and the proportion of early Implanon discontinuation of 23.4% (18).

The calculated sample size (*n*) is


$$n\;=\;\frac{\left(1.96{)}^{2}x\;0.234\;(1\;-\;0.234\right)}{0.05^{2}}\;=\;275$$

Considering for a 1.5 design effect and a non-response rate of 10%, the sample size was 454 women who discontinued Implanon.

### Sampling procedure

**Figure 1:**
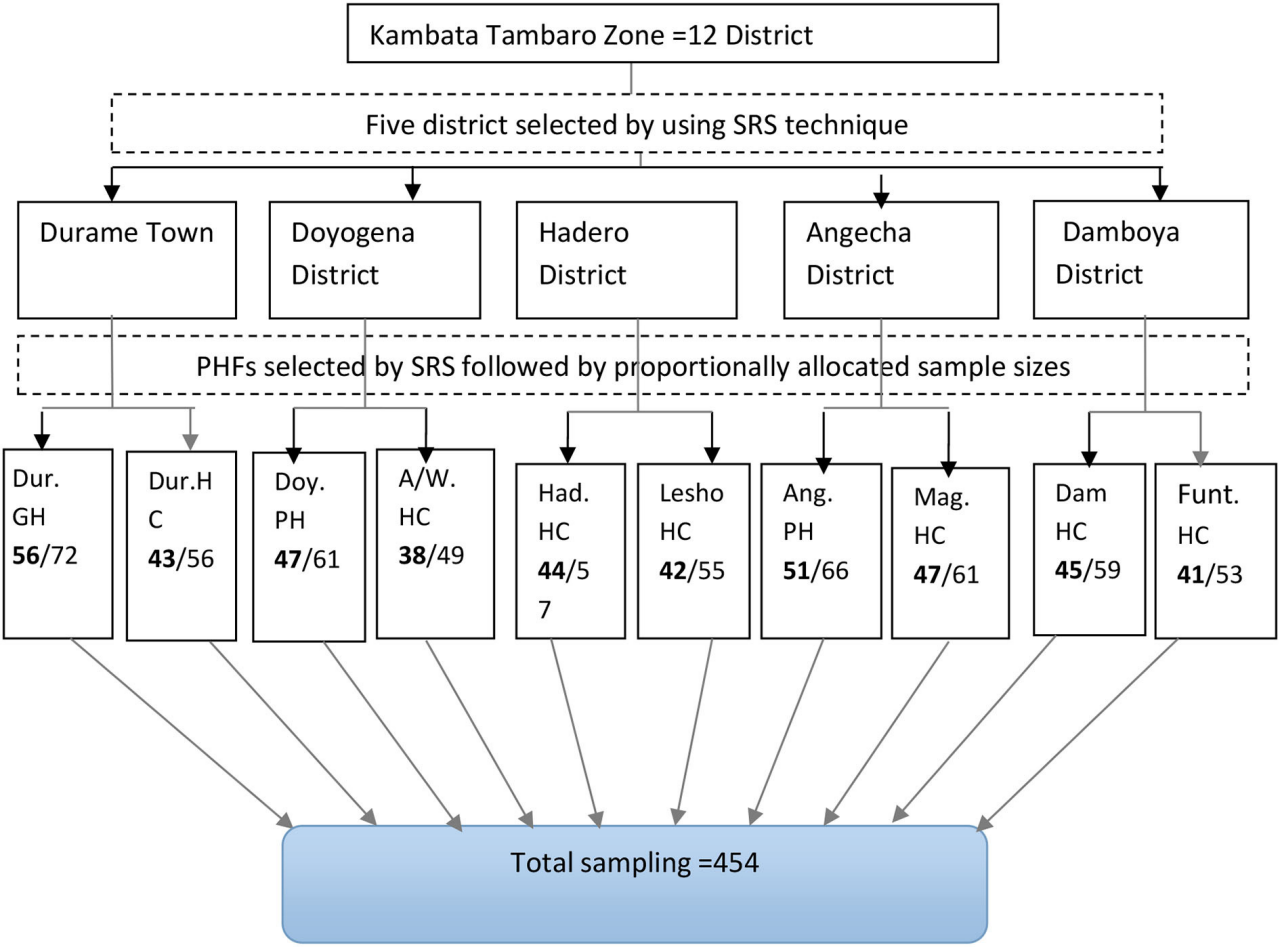
Schematic presentation of months of sampling procedure among Implanon users visiting public health facilities to discontinue Implanon in the Kambata Tambaro zone, 2020. GH, General hospital; HC, Health center; PH, Primary hospital; PHF, Public health facilities; SRS, Simple random sampling.

The study participants were sampled using a multi-stage sampling technique. The simple random sampling (lottery) method was used to select five districts from the 12 districts in the Kembata zone. Out of five selected district ten health facilities two from each district were selected randomly. The final sample size was proportionally allocated to the selected health facilities, and study participants were enrolled by a simple random sampling technique (Figure 1).

### Data collection

A face-to-face interview was used to collect data using a pre-tested and structured questionnaire. The data collection tool was developed based on family planning indicators from the Ethiopian Demographic Health Survey 2016 and variables collected from different relevant literature works (15, 19, 20). It contains the sociodemographic background of women, the history of obstetrics, and the history of the use of contraceptive methods. The questionnaires were initially prepared in English and then translated into Amharic and Kambatisa and later backtranslated into English by language experts to check its consistency. Data collection was carried out by 8 female diploma midwives who were fluent speakers of the 2 languages (Amharic and Kambatisa). In addition two bachelor degree holder midwifery were recruited to supervise the data collection.

### Variables

#### Dependent variable

Early Implanon discontinuation.

#### Independent variables

The sociodemographic character of the study participant, obstetric-related characteristics, and contraceptive method-related characteristics are independent variables.

### Operational definition

Early Implanon discontinuation is defined as the removal of Implanon before completing 2.5 years since its insertion (8, 21). The date of insertion was taken from medical records and their appointment card. Then, the date is counted from insertion to discontinuation.

### Data quality control

To ensure data quality, the questionnaires were pretested on 5% of the total sample size at Serera Health Center. The questionnaires were modified based on the pretest result, repetitive ideas and ambiguous questions were corrected, and the modified questionnaire was used for the final data collection. Data collectors and supervisors received two days of training on data collection and interview techniques. Proper categorization and coding of questionnaires were critically prepared before the data collection. Moreover, the supervisors and the principal investigator carefully checked the collected data daily for completeness, accuracy, and clarity.

### Data processing and analysis

After data collection, the questionnaires were checked for its completeness, cleaned up, and coded. Then, data entry was performed using Epi Data version-3.1 and exported to STATA version-16 software for analysis. Simple frequencies and percentages were used to summarize data on the magnitude of early Implanon discontinuation. A bivariate logistic regression analysis was performed to examine the association between each independent variable and the outcome variable. Based on the findings, variables with a *p* < 0.2 in the bivariate logistic regression analysis were included in the multivariable logistic regression analysis. The adjusted odds ratio (AOR) along with the 95% confidence interval was estimated to assess the strength of the association. Variables with a *p* < 0.05 in the multivariable logistic regression analysis were considered as significant and independent predictors of early Implanon discontinuation. Hosmer and Lemeshow goodness-of-fit was used to assess whether the necessary assumptions were met. The model was considered to be well suited because it was not significant for the Hosmer–Lemeshow statistic (*p* = 0.610). A multicollinearity test was performed to see the correlation among the independent variables using collinearity statistics [variance inflation factor (VIF)], and no variables with a VIF >10 or a tolerance test < 0.1 were observed. Finally, the information was presented in the form of statements, tables, and graphs.

### Ethical considerations

The present study was reviewed and approved by the Institutional Health Research Ethics Review Committee of the Colleges of Health and Medical Sciences, Haramaya University. The study participants were informed that their responses were kept confidential throughout the research process. For study participants who were less than 18 years, consent was obtained from their family or legal guardians. Data collection began after obtaining informed, voluntary written consent from each study participant.

## Results

### Sociodemographic characteristics of study participants

In the present study, 438 women visiting selected public health facilities in the zone were interviewed, resulting in a response rate of 96.48%. The age of the study participants ranged from 17 to 43 years, with the mean [±standard deviation (SD)] age of 26.88 ± 5.06 years. In addition, around 244 (55.7%) of the study participants lived in rural areas. Regarding educational status, 129 (29.5%) were women and 162 (38.8%) had husbands who had a college degree or higher. The result showed that half of the 222 (50.7%) study participants were housewives (Table 1).

**Table 1:** Socio-demographic characteristics of Implanon user women visiting public health facilities to discontinue Implanon in Kembata Tambaro zone, South Ethiopia, 2020 (*n* = 438).

**Variables**	**Category**	**Frequency**	**Percent**
Age of women	15–19	23	5.3
	20–24	113	25.8
	25–29	159	36.3
	30–34	103	23.5
	≥35	40	9.1
Marital status	Married	417	95.2
	Single	9	2.1
	Divorced	4	.9
	Widowed	8	1.8
Women religion	Protestant	348	79.5
	Orthodox	78	17.8
	Others	12	2.7
Ethnicity	Kambata	358	81.7
	Hadiya	50	11.5
	Others	30	6.8
Women occupation	Government employee	125	28.5
	Merchant	60	13.7
	House wife	222	50.7
	Student	26	5.9
	Other	5	1.1
Women education	Can't read and write	40	9.1
	Can read and write	91	20.8
	Primary (1–8)	117	26.7
	Secondary (9–12)	61	13.9
	diploma and above	129	29.5
Husband's education (*n* = 417)	Can't read and write	18	4.3
	Can read and write	59	14.1
	Primary (1–8)	86	20.6
	Secondary (9–12)	92	22.1
	diploma and above	162	38.8
Residence of woman	Urban	194	44.3
	Rural	244	55.7

### Obstetric-related characteristics

Regarding the parity of the study participants, 164 (37.4%) respondents had given birth three to four times. Around 325 (74.2%) of the women had at least one living child. In addition, 98 (22.4%) of the women had experienced a miscarriage in their previous pregnancy. Furthermore, 50% of the study participants had a desire to have a child soon, while 104 (47.1%) of them desire to have a child within 2 years (Table 2).

**Table 2:** Pregnancy and related characteristics of Implanon users women visiting public health facilities to discontinue Implanon in Kembata Tambaro zone, South Ethiopia, 2020 (*n* = 438).

**Variables**	**Category**	**Frequency**	**Percentage**
Parity	0	26	5.9
	1–2	154	35.2
	3–4	164	37.4
	≥5	94	21.5
Number of alive children	0	29	6.6
	1–2	156	35.6
	3–4	169	38.6
	≥5	84	19.2
Abortion	Yes	98	22.4
	No	340	77.4
Types of abortion (*n* = 99)	Spontaneous	79	79.8
	Induced	20	20.2
Desire for pregnancy	Yes	221	50.5
	No	217	49.5
Time for pregnancy desire (*n* = 221)	Within 2 years	104	47.1
	After 2 years	51	23.1
	Not decide time	66	29.9

### Utilization of contraceptive methods and other related characteristics

Of the total study participants, 359 (82%) had used at least one modern contraceptive before undergoing current Implanon removal. The most commonly used modern contraception methods by these participants were injectable 231 (64.3%) and Pills 131 (36.5) before inserting Implanon. More than half (51.6%) of the participants received Implanon insertion services from the hospital, while 183 (41.8%) and 29 (6.6%) women received services from health centers and health posts, respectively. On the contrary, 380 (86.8%) participants indicated that they were counseled about the benefits and side effects of Implanon during the insertion period. In addition 353(80.5%) of women had support from their husband to use contraceptive. Furthermore, 326 (74.4%) of the study participants reported that they were satisfied with the service provided during Implanon insertion (Table 3).

**Table 3:** Contraceptive method utilization and related characteristics among women visiting public health facilities to discontinue implanon in Kembata Tambaro Zone southern Ethiopia, 2020 (*n* = 438).

**Variables**	**Category**	**Frequency**	**Percentage**
History of other modern contraceptive uses	Yes	359	82.0
	No	79	18.0
Types of contraceptive used before (*n* = 359)	OCP	131	36.5
	Injectable	231	64.3
	Jadelle	48	13.4
	IUCD	18	5
	Others	2	0.6
Counseled about Implanon benefits	Yes	380	86.8
	No	58	13.2
Counseled about Implanon side effects	Yes	380	86.8
	No	47	13.2
Ever faced side effects of Implanon	Yes	181	41.3
	No	257	58.7
Types of side effect ever faced (more than one choice)	Heavy menstrual bleeding	88	48.6
	Weight change	24	13.3
	Unusual headache	45	24.9
	Insertion site pain	28	15.5
	Blurring of vision	36	19.9
	Others	5	2.8
Implanon choose	Own choice	278	63.5
	Husband	106	24.2
	HEW	16	3.7
	Health provider	26	5.9
	Others	12	2.7
Service satisfaction	Yes	326	74.4
	No	112	25.6
Follow-up appointment	Yes	348	79.5
	No	90	20.5

### The magnitude of early Implanon discontinuation

**Figure 2:**
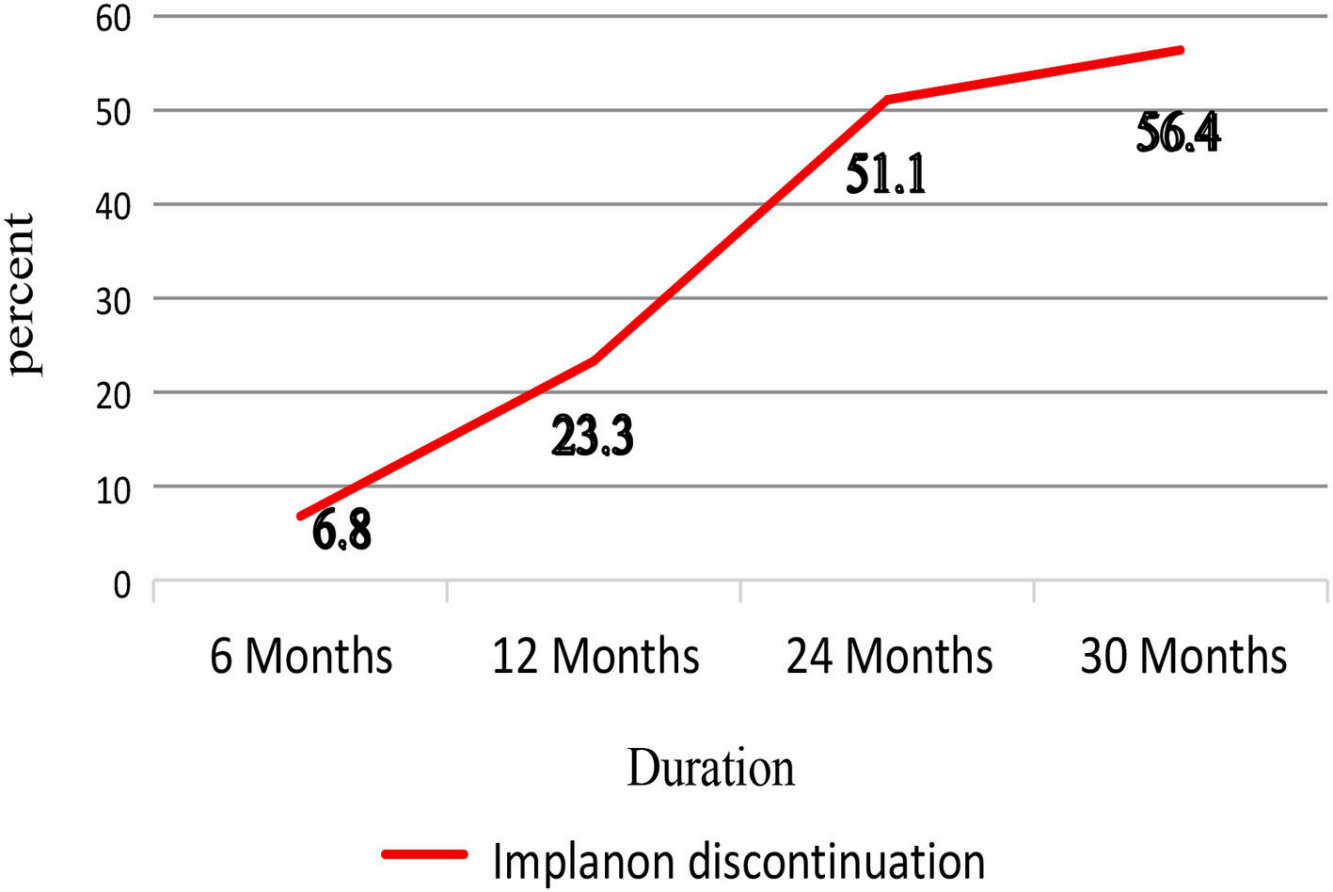
Magnitude of early Implanon discontinuation among implanon users women visiting public health facilities to discontinue Implanon in Kembata Tambaro zone, South Ethiopia, 2020 (*n* = 438).

In the present study, early Implanon discontinuation was reported by 247 women who discontinued Implanon at the public health facilities in the Kambata zone. This corresponds to a proportion of 56.4%, 95% CI (51.6–61.2), of which 6.8 and 23.3% discontinued before 6 and 12 months, respectively (Figure 2).

### Reasons for early Implanon discontinuation

**Figure 3:**
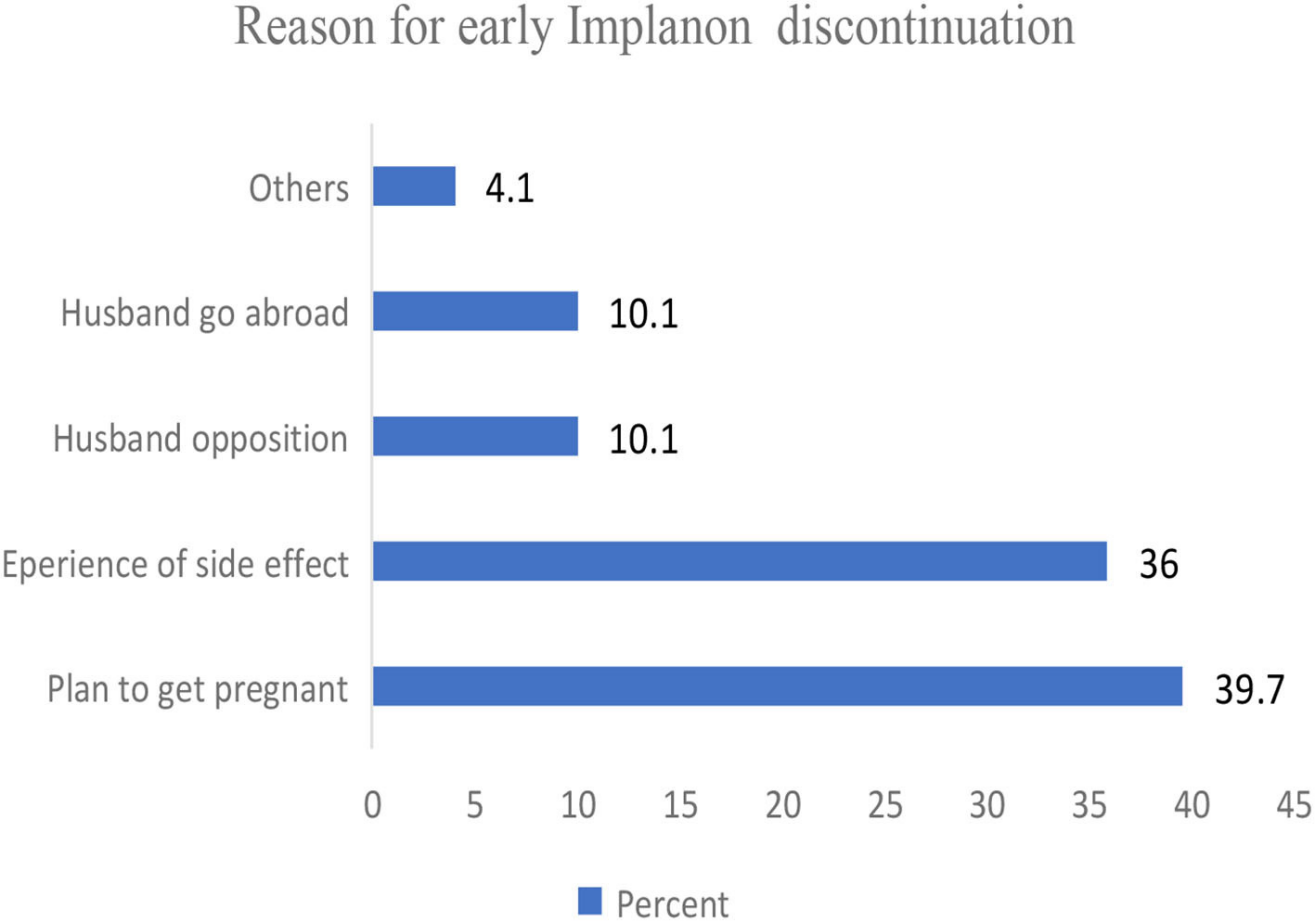
Reason for early Implanon discontinuation among Implanon users women visiting public health facilities to discontinue Implanon in Kembata Tambaro zone, south Ethiopia, 2020 (*n* = 438).

In the study, 39.7% of the reasons stated for early Implanon discontinuation were becoming pregnant and having side effects due to Implanon and 36% for other reasons (death of husband, divorce, and method shift) (Figure 3).

### Factors associated with early Implanon discontinuation

The relationship between each independent variable and the outcome variable was analyzed separately. In the bivariate analysis, variables like women's education, the place of residence, history of abortion, husband's support, counseling on the side effects of Implanon, counseling on the benefits of Implanon, those who developed side effects, satisfaction with the service given, and follow-up appointments showed an association with early Implanon discontinuation at a *p* ≤ 0.2.

Multivariable logistic regression was performed to control for confounding variables and to identify factors associated with early Implanon discontinuation. Thus far, women living in rural areas (AOR = 1.76, 95% CI: 1.06–2.92), women having primary educational status [AOR = 2.92, 95% CI (1.56, 5.46)], woman who had no history of contraceptive use [AOR = 2.14, 95% CI (1.14, 4.03)], women who experienced side effects [AOR = 3.36, 95% CI (2.09, 5.42)], those who lack follow-up for a pre-insertion appointment [AOR = 2.17, 95% CI (1.18, 4.79)], and those dissatisfied with services [AOR = 3.05, 95% CI (1.52, 6.12)] were associated with early Implanon discontinuation at a *p* < 0.05 (Table 4).

**Table 4:** Factors associated with Early Implanon discontinuation among implanon users women visiting public health facilities to discontinue Implanon in Kembata Tambaro zone, South Ethiopia, 2020 (*n* = 438).

**Variables**	**Category**	**Early Implanon discontinuation**		**COR (95%CI)**	**AOR (95%CI)**
		**Yes (%)**	**No (%)**		
Women education	Cannot read and write	27 (67.5)	13 (32.5)	3.51 (1.65–7.43)	2.29 (0.93–5.65)
	Can read and write	64 (70)	27 (30)	3.97 (2.25–7.10)	3.45 (1.69–6.99)**
	Primary	75 (64)	42 (36)	3.0 (1.79–5.07)	2.92 (1.56–5.46)*
	Secondary	33 (54)	28 (46)	1.99 (1.07–3.69)	1.83 (0.87–3.83)
	Diploma and above	48 (37)	81 (63)	1	1
Residence	Urban	90 (46.4)	104 (53.6)	1	1
	Rural	157 (64.3)	87 (35.7)	2.09 (1.42–3.07)	1.76 (1.06–2.92)*
Abortion history	Yes	69 (70)	29 (30)	2.17 (1.34–3.51)	1.24 (0.70–2.19)
	No	178 (52.3)	162 (47.7)	1	1
History of modern contraceptive use	Yes	195 (54)	164 (46)	1	1
	No	52 (66)	27 (34)	1.62 (0.97–2.70)	2.14 (1.14–4.03)*
Husband support to use contraceptive method	Yes	193 (54.7)	160 (45.3)	1	1
	No	54 (64.6)	31 (36.4)	1.47 (0.91–2.76)	0.89 (0.45–1.75)
Ever faced Implanon side effect	Yes	133 (73.5)	48 (26.5)	3.48 (2.3–4.25)	3.36 (2.09–5.42)**
	No	114 (44.4)	143 (55.6)	1	1
Counseled on Benefit of Implanon	Yes	206 (54.2)	174 (45.8)	1	1
	No	41 (70.7)	17 (29.3)	2.04 (1.12–3.71)*	0.33 (0.07–1.55)
Counseled on Side effect of Implanon	Yes	204 (53.7)	176 (56.3)	1	1
	No	43 (74.1)	15 (25.9)	2.47 (1.33–4.60)*	3.69 (0.76–17.78)
Satisfied with the service	Yes	155 (47.6)	171 (52.4)	1	1
	No	92 (82.1)	20 (17.9)	5.08 (2.99–8.62)**	3.05 (1.52–6.12)*
Appointed for follow-up after insertion	Yes	172 (49.4)	176 (50.6)	1	1
	No	75 (83.3)	15 (16.7)	5.12 (2.83–9.26)**	2.17 (1.18–4.79)*

OCP, oral contraceptive pills; HEW, health extension worker; *p < 0.05 and **p < 0.001. Bold values indicate the referent values.

## Discussion

The current study attempts to determine the magnitude of early Implanon discontinuation and associated factors in the study area. Thus, the study indicated that early Implanon discontinuation is high in number. In addition, factors like educational status, place of residence, counseling on the side effects of Implanon, lack of follow-up appointment after the insertion of Implanon, and lack of satisfaction with the service provided were associated with early Implanon discontinuation.

The current study revealed that around 56.4% of women discontinued Implanon early. The result of the present study is consistent with the study conducted in Queensland, Australia, in which Implanon discontinuation was observed within 2.5 years (57.7%) (22). However, this finding is higher than that of studies conducted in India (37%), Yemen (43%), and central Ethiopia (46.3%) (23–25). In addition, the result of this study was lower than that of the studies conducted in the town of Debre Tabor and in the district of Andebet of north-western Ethiopia, which reported the magnitude of early Implanon discontinuation as 65 and 85%, respectively (19, 26). This discrepancy could be attributed to sociocultural and country differences in the coverage and quality of reproductive health services. Furthermore, because this study was institutional and some of the studies used for comparison were conducted at the community level, the disparity in sample size and study setting could account for this difference.

The findings from this study showed that the main reason of women who requested early Implanon discontinuation was suffering from side effects and their husband's resistance. This finding was also reported in the study conducted in the Andebet district in north-western Ethiopia (19). This could be due to inadequate pre-insertion counseling on the side effects of Implanon as the side effects of Implanon, such as menstrual disruption, can disrupt their daily activities and sexual relations with their spouse. Thus, involving the husband in the discussion during insertion is a good solution to maintain this contraceptive method until the removal due date.

However, this study found that women with primary educational status were 2.9 times more likely to discontinue Implanon early than women with college or higher educational status. The findings from the current study were found to be consistent with the findings from other studies conducted in Bangladesh, South Africa, Kenya, and Mekelle, which found that women with higher levels of education were less likely to discontinue Implanon before the due date (5, 16, 27, 28). One possible explanation might be that women with higher levels of education understand the benefits of contraceptives and their side effects better, which help them to handle and maintain Implanon.

In contrast, the present study showed that women who reside in rural areas were 1.76 times more likely to discontinue Implanon early compared to those who reside in urban areas. This is supported by a study conducted in Bangladesh, which reported that rural communities were more likely to discontinue Implanon than those living in urban communities (27). Women who live in rural areas are likely to lack access to information and use reproductive services as early as they would like, which may lead them to remove Implanon early due to mild side effects.

Moreover, this study found that women who had no history of modern contraceptive use before the current Implanon were 2.1 times more likely to discontinue Implanon early than those who had a history of contraceptive use. This could be because women who previously used contraceptive methods had a better understanding of how to cope with the side effects. The finding of the present study was supported by a study conducted in the Tigray Region, which revealed that women with a history of contraceptive use were 11 times less likely to discontinue Implanon compared to women with no history of contraceptive use (29).

Furthermore, the current study revealed that women who experienced the side effects of Implanon were 3.3 times more likely to discontinue Implanon early because the side effects of Implanon, such as menstrual disruption, can interfere with their daily activity and sexual life. This agrees is in line with studies conducted in India (23), the USA (30), Tigray (31), and the city of Hawasa (32). As a result, comprehensive counseling on the side effects of Implanon during Implanon insertion, with a focus on how to cope with the side effects, is beneficial in overcoming early Implanon discontinuation due to the side effects of Implanon (33).

To crown up, the findings of this study also showed that women who were not satisfied with the service provided during the insertion of Implanon were three times more likely to discontinue Implanon early than those who were satisfied. This could be due to a lack of privacy and improper pre-insertion counseling on expected side effects of Implanon, which can lead to women being dissatisfied with the service and contributing to early Implanon discontinuation. This finding is consistent with a study conducted in southern Ethiopia (18). Therefore, providing quality reproductive health services to clients can lead to better satisfaction and use of the services (34).

## Conclusion

The magnitude of early Implanon discontinuation was high. Educational status, lack of a previous history of contraceptive method usage, women who experienced side effects, rural residents, those dissatisfied with the service provided, and a lack of follow-up appointments. Improving partner involvement in family planning services (decision-making) can increase the continuation rate of Implanon contraceptives among user women. Healthcare providers must focus on pre-insertion counseling on Implanon side effects, which is essential in tackling discontinuation related to Implanon side effects. In addition, early management of side effects and reassurance are essential to minimize early Implanon discontinuation. Furthermore, improving client service satisfaction and appointment follow-up also should be considered to escalate the proper use of Implanon.

## Limitations and strengths of this study

In the present study, the magnitude of early Implanon discontinuation was assessed using an appropriate sample size. This study addressed cultural issues, including female data collection, as it is common in the community to discuss reproductive issues with the same gender. This may also reduce the social desirability bias. This study is based on one facility; it may not indicate the true early Implanon discontinuation in the community. A limitation of this study is that women without an appointment card may have had difficulty remembering the insertion date of Implanon (recall bias).

## Data availability statement

The raw data supporting the conclusions of this article will be made available by the authors, without undue reservation.

## Ethics statement

The studies involving human participants were reviewed and approved by Institutional Health Research Ethics Review Committee of the Colleges of Health and Medical Sciences, Haramaya University. Written informed consent to participate in this study was provided by the participants' legal guardian/next of kin.

## Author contributions

GB was the principal investigator who initiated the research, participated in writing the proposal, formal conducted data analysis, and participated in writing the original draft of this manuscript. NA, TY, TM, and HBE provided general guidance for overall study progress and participated in proposal review, analysis review, and manuscript writing. All authors have read and approved the final manuscript and are accountable for all aspects of this work.

## Funding

Doyogena primary hospital has provided financial support for this study. The authors declared that the funding body has no role in designing the study, data collection, data analysis, and writing the manuscript.

## Conflict of interest

The authors declare that the research was conducted in the absence of any commercial or financial relationships that could be construed as a potential conflict of interest.

## Publisher's note

All claims expressed in this article are solely those of the authors and do not necessarily represent those of their affiliated organizations, or those of the publisher, the editors and the reviewers. Any product that may be evaluated in this article, or claim that may be made by its manufacturer, is not guaranteed or endorsed by the publisher.
